# Knowledge and attitudes of health science students and medical interns at al Al-Baha University towards epistaxis first aid

**DOI:** 10.25122/jml-2024-0218

**Published:** 2024-12

**Authors:** Rajab Alzahrani, Wafaa Taishan, Mujtaba Ali, Alshaik Tarek, Alahmadi Khalid, Abdulrahman Almaymoni, Renad Alshehri, Lamyaa Almathahibi, Ahmed Khuzayyim, Ibrahim Al Suliman, Mohammed Alabbadi, Dalal Alghamdi

**Affiliations:** 1 Department of Surgery, Faculty of Medicine, Al-Baha University, Al-Baha, Saudi Arabia; 2 Faculty of Medicine, Al-Baha University, Al-Baha, Saudi Arabia; 3 King Fahad Hospital, 2nd Cluster Ministry of Health, Jeddah, Saudi Arabia; 4 East Jeddah Hospital 1st Cluster, Jeddah, Saudi Arabia; 5 King Khaled University, Abha, Saudi Arabia; 6 Najran University, Najran, Saudi Arabia; 7 King Faisal University, Hofuf, Saudi Arabia

**Keywords:** epistaxis, prevalence, medicine, hemorrhage, Saudia Arabia

## Abstract

Epistaxis, or nosebleeds, is a common medical concern in emergency departments worldwide, often triggered by trauma, infections, allergies, and high blood pressure. Despite its frequency, there is limited research on the knowledge and attitudes of health-related students regarding the first aid management of epistaxis. This study aimed to assess the knowledge and attitudes toward first aid management of epistaxis among health-related specialty students at Al-Baha University, Saudi Arabia. A cross-sectional study was conducted using a self-administered online questionnaire. A total of 370 participants were recruited through convenience sampling. Data were analyzed using descriptive statistics and chi-square tests to identify significant associations. The findings revealed that over 70% of participants showed strong knowledge in identifying causes of epistaxis and applying first aid, although only 9.6% correctly differentiated between various causes of nosebleeds. Attitudes were highly positive, with 92.1% of respondents supporting training in nosebleed first aid and 85.9% recognizing epistaxis as a health priority. Significant differences in knowledge scores were observed based on age, academic year, and previous training (*P* < 0.05). Participants reported a median self-assessed confidence and knowledge rating of 6 on a 10-point scale. These findings underscore the need for targeted education and training in nosebleed management among healthcare students. The study highlights the need to address knowledge gaps and improve attitudes towards epistaxis first aid management among health-related specialty students at Al-Baha University in Saudi Arabia.

## INTRODUCTION

Epistaxis, commonly referred to as a nosebleed, is a medical concern frequently presenting in emergency departments (EDs) due to its sudden onset and potential severity. Various factors, such as trauma, infections, allergies, and high blood pressure, can function as local, systemic, or environmental triggers to cause epistaxis [1]. It is one of the most common ear, nose, and throat (ENT) emergencies encountered globally [2]. Though uncommon in infants and young children, it is a prevalent disorder in children and young adults [3]. Epistaxis is considered a potentially life-threatening rhinological emergency, with bleeding severity ranging from mild to severe. It contributes significantly to the workload in accident and emergency (A&E) and otolaryngology departments, often causing anxiety for both patients and healthcare professionals [4].Studies report that 10% to 60% of individuals experience at least one major episode of epistaxis during their lifetime [5].

While aggressive intervention and hospitalization may be necessary for some epistaxis events, most patients respond well to basic first aid procedures [6]. First aid is the early and basic care an unskilled or inexperienced person gives to an injury or illness until medical attention can be obtained [7]. Knowledge of first aid is essential to education, particularly for students in health-related professions. Although these students receive training to handle emergencies in hospital settings with access to necessary medications and resources [8], their knowledge and preparedness to manage emergencies outside hospital environments, such as at the scene, may be insufficient [9,10]. Despite numerous studies on epistaxis, there is a lack of documentation on the knowledge and attitudes of health-related specialty students regarding first aid management of epistaxis [10]. Accordingly, this study aimed to assess and promote adequate knowledge and attitudes regarding first aid management of epistaxis among health-related specialties students at Al-Baha University, Saudi Arabia.

## MATERIAL AND METHODS

### Study design

A cross-sectional survey was conducted between March 3^rd^ and April 6^th^, 2024, at Al-Baha University, Saudi Arabia. The study focused on health-related specialty students, including those from medicine, pharmacy, and health sciences programs.

### Inclusion and exclusion criteria

The study included male and female students and interns from health-related specialties (medicine, pharmacy, and health sciences) at Al-Baha University who agreed to participate. We excluded non-health-related specialties students, health-related specialties, and medical interns from other universities, as well as participants with incomplete questionnaires.

### Sample size calculation

The sample size was determined using Cochran’s equation, with a precision level of ±5% and a confidence level of 95%. Based on an estimated total population of 10,000 health-related specialty students and interns, the calculated sample size was 370 participants.

### Data collection

Data were collected using an anonymous, self-administered electronic questionnaire in English and Arabic. The questionnaire was distributed to health-related specialty students and interns via Telegram and WhatsApp. Prior to participation, all individuals were informed about the study's objectives and assured of data confidentiality. The questionnaire included demographic information, such as age, gender, specialty, clinical year, residency, and sections assessing epistaxis knowledge and attitudes. A pilot study with 20 participants was conducted to evaluate the clarity and suitability of the questionnaire and estimate the time required for data collection.

### Statistical analysis

IBM SPSS version 27.0.1 was employed to analyze the data. Descriptive statistics were utilized to summarize the study participants’ characteristics and responses. Frequencies and percentages were calculated for categorical variables, such as sociodemographic characteristics and responses to knowledge and attitude questions. For continuous variables, medians and interquartile ranges (IQRs) were reported. Participants responded to the questions regarding knowledge and attitude towards first aid management on a scale ranging from 'strongly agree' to 'strongly disagree', with each response option corresponding to a numerical score: strongly agree (4), agree (3), uncertain (2), disagree (1) and strongly disagree (0). The total scores for knowledge and attitude ranged from 0 (minimum) to 28 (maximum). To compare knowledge and attitude scores among different groups, Mann-Whitney U tests, or Kruskal-Wallis tests were conducted. These non-parametric tests were chosen due to the non-normal distribution of the scores, as assessed by the Kolmogorov-Smirnov Test (*P* < 0.05). Median scores, IQRs, and *P* values from the tests were calculated to determine statistically significant differences among groups. A significance level of *P* < 0.05 was used for all statistical tests, indicating a 95% confidence interval. Of 378 potential participants, five declined participation, and three were from non-health-related specialties. Therefore, the analysis included 370 cases.

## RESULTS

The sociodemographic characteristics of the participants (*n* = 370) are detailed in Table 1. Gender distribution revealed that most participants were women, accounting for 252 individuals (68.1%), with male participants constituting 118 individuals (31.9%). Regarding the academic year, the distribution shows a relatively even representation across different stages of education. Notably, most participants were in their internship year (*n* = 73, 19.7%), followed closely by those in their fifth and sixth years, each accounting for 56 individuals (15.1%). The field of study varied among participants, with the majority majoring in medicine (*n* = 215, 58.1%), followed by nursing (*n* = 62, 16.8%), pharmacy (*n* = 43, 11.6%), allied health sciences (*n* = 30, 8.1%), dentistry (*n* = 8, 2.2%), and other (*n* = 12, 3.2%). Additionally, 146 participants (39.5%) received formal training or education in nosebleed first aid management during their academic program (Table 1).

**Table 1:** Sociodemographic characteristics of participants (*n* = 370)

Variable	n	(%)
**Gender**		
Female	252	(68.1%)
Male	118	(31.9%)
**Age (years)**		
18 - 22	186	(50.3%)
23 - 27	160	(43.2%)
28 - 32	9	(2.4%)
33 years or older	15	(4.1%)
**Academic year**		
First-year	36	(9.7%)
Second year	48	(13.0%)
Third year	50	(13.5%)
Fourth-year	51	(13.8%)
Fifth year	56	(15.1%)
Sixth year	56	(15.1%)
Internship year	73	(19.7%)
**Field of study**		
Allied health sciences	30	(8.1%)
Dentistry	8	(2.2%)
Medicine	215	(58.1%)
Nursing	62	(16.8%)
Pharmacy	43	(11.6%)
Training or education in nosebleed first aid management	146	(39.5%)
Others		
NB	12	(3.2%)

*n*, Frequency; %, Percentage

The analysis of participants' responses in Table 2 reveals several key findings regarding their knowledge and attitudes toward nosebleed management. In terms of knowledge assessment, a significant portion of participants demonstrated a firm grasp of essential concepts, with over 70% indicating their ability to identify common causes of nosebleeds, and a majority of participants were familiar with the anatomy of the nasal cavity. Additionally, a substantial majority expressed confidence in their ability to apply first aid techniques and were aware of potential complications associated with untreated nosebleeds. However, fewer participants understood specific concepts, such as the difference between anterior and posterior nosebleeds. Regarding attitudes, the majority of participants recognized the importance of healthcare students receiving training in emergency first aid for nosebleeds and believed in the significance of nosebleeds as a health issue requiring prompt attention. Moreover, there was strong agreement on the need for ongoing education and training for healthcare workers in managing common medical emergencies like nosebleeds.

**Table 2:** Response of participants regarding knowledge and attitude assessment

	Disagree	Neutral	Agree
	*n* (%)	*n* (%)	*n* (%)
I can identify common causes of nosebleeds	36 (9.8%)	58 (15.7%)	276 (74.6%)
I am familiar with the anatomy of the nasal cavity and the structures involved in nosebleeds	65 (17.6%)	83 (22.4%)	222 (60%)
I know the appropriate emergency procedures to deal with nosebleeds	43 (11.6%)	47 (12.7%)	280 (75.7%)
I am confident in my ability to apply first-aid techniques to control nosebleeds	51 (13.8%)	61 (16.5%)	258 (69.8%)
I am aware of the potential complications associated with untreated or improperly managed nosebleeds	67 (18.1%)	68 (18.4%)	235 (63.5%)
I understand the difference between anterior and posterior nosebleeds and the proper ways to manage them	128 (34.6%)	72 (19.5%)	170 (46%)
I know when it is appropriate to seek medical help for a nosebleed after taking basic first-aid measures	70 (19%)	58 (15.7%)	242 (65.4%)
I think it is important for healthcare students to have training in emergency first aid for dealing with nosebleeds	9 (2.5%)	20 (5.4%)	341 (92.1%)
I feel comfortable providing first aid assistance to someone with a nosebleed	29 (7.8%)	44 (11.9%)	297 (80.3%)
I believe that nosebleeds are an important health problem that requires prompt attention	10 (2.7%)	42 (11.4%)	318 (85.9%)
I believe that improving knowledge about nosebleed management among healthcare students can positively impact patient outcomes	6 (1.6%)	26 (7.0%)	338 (91.4%)
I am interested in participating in workshops or training sessions focused on nosebleed care	33 (8.9%)	61 (16.5%)	276 (74.6%)
I believe that healthcare workers should receive ongoing education and training in managing common medical emergencies such as nosebleeds	7 (1.9%)	23 (6.2%)	340 (91.9%)
I think there is a need for more public awareness campaigns on how to react effectively in case of nosebleeds	7(1.9%)	29 (7.8%)	334 (90.3%)

*n*, Frequency; %, Percentage

Table 3 compares total knowledge and attitude scores among different participant groups. Women scored higher on median knowledge and attitude, with knowledge categorized as 'fair' (50–75% quartile) and attitudes as 'good' (>75% quartile). Men scored in the < 50% quartile for knowledge, but these differences were not statistically significant (*P* = 0.066 for knowledge, *P* = 0.229 for attitude). Younger participants (18–22 years) had significantly lower knowledge scores (< 50% quartile) compared to older groups (*P* < 0.001). However, no significant differences were found in attitude scores by age (*P* = 0.152). Regarding academic year, first-year students had significantly lower median total knowledge scores (< 50% quartile) compared to other academic years (*P* < 0.001), while no significant difference was observed in attitude scores among different academic years (*P* = 0.929). Finally, participants who received formal training or education in nosebleed first aid management demonstrated significantly higher median total knowledge scores in the 50–75% quartile compared to those who did not receive such training (*P* < 0.001), with no significant difference in attitude scores (*P* = 0.515).

**Table 3:** Comparison of total knowledge and attitude scores among various characteristics

Variables	Total knowledge score	Sig^1^	Total attitude score	Sig^1^
	*n*, Median (%)		*n* Median (%)	
**Gender**				
Women	252 (50-75 %)	0.066	252 (>75%)	0.229
Men	118 (<50%)		118 (50-75%)	
**Age (years)**				
18 - 22	186 (< 50%)	0.005*	186 (>75%)	0.152
23 - 27	160 (50-75%)		160 (>75%)	
28 - 32	9 (50-75%)		9 (>75%)	
≥33	15 (50-75%)		15 (>75%)	
**Academic year**				
First	36 (< 50 %)	0.001*	36 (>75%)	0.929
Second	48 (< 50%)		48 (>75%)	
Third	50 (50-75%)		50 (>75%)	
Fourth	51 (50-75%)		51 (>75%)	
Fifth	56 (>75%)		56 (>75%)	
Sixth	56 (>75%)		56 (>75%)	
Internship	73 (50-75%)		73(>75%)	
**Field of study**				
Allied health sciences	30 (< 50%)	0.101	30 (>75%)	0.516
Dentistry	8 (>75%)		8 (>75%)	
Medicine	215 (50-75%)		215 (>75%)	
Nursing	62 (50-75%)		62 (>75%)	
Pharmacy	43 (< 50%)		43 (>75%)	
Other	12 (< 50%)		12 (>75%)	
**Previous training or education in nosebleed first aid management**				
Yes	146 (50-75%)	0.001*	146 (>75%)	0.515
No	224 (< 50%)		224 (>75%)	

^1^*P* value , *P <0.05, significant, *n*: Frequency, %, Percentage

Table 4 concluded that the majority of participants (50%) demonstrated a moderate level of confidence in their first-aid management of nosebleeds. This is evident from the median score 6.00, which falls within the second quartile (50–75% range). Only 25% of participants expressed high confidence (scores 8–10), while the remaining 25% displayed low confidence (scores 1–4). These findings highlight a need for further education and training to improve participants' confidence and knowledge.

**Table 4:** Self-assessment of participants

	Median (IQR) (%)
On a scale of 1 to 10, how confident are you that you could effectively deal with a nosebleed using first aid techniques?	6 (50-75%)
How would you rate your general knowledge level regarding first aid management of nosebleeds?	6 (50-75%)

## DISCUSSION

Our study revealed that most participants had a strong understanding of nosebleed management, with over 70% identifying common causes and being familiar with nasal cavity anatomy. Participants expressed confidence in applying first-aid techniques and were aware of potential complications. However, fewer participants understood specific concepts like anterior and posterior nosebleeds. Compared to our findings, a similar study reported that 85.6% of participants experienced or observed an episode of epistaxis, with 64% considering it an emergency. The most common causes identified were fingernail trauma, followed by bleeding disorder (17.3%), hypertension (14.3%), and nasal fracture (5.3%) [11]. Albouq *et al*. [12] revealed that 87.1% of respondents believed bleeding disorder was the primary cause of epistaxis. Compared to our study, another study reported that 81.4% of participants experienced epistaxis and attempted to manage it by applying pressure. Among these, 20.7% applied pressure to the lower portion of the nose, 36.5% to the upper portion, and 42.8% were unaware of the correct pressure application site. During an episode of epistaxis, 64.9% of respondents leaned their heads backward, an incorrect practice [13]. Consistent with our findings, a different study found that the majority of participants (56.9%) thought that the best body position to stop nose bleeding was to lean forward. However, 36.5% still believed leaning backward was appropriate, while smaller proportions suggested lying on the back (5.1%) or the abdomen (5.1%) [14].

Regarding attitudes in our study, 69.7% of participants recognized the importance of healthcare students receiving training in emergency first aid for nosebleeds and believed in the significance of nosebleeds as a health issue requiring prompt attention. Moreover, 61.4% agreed on the need for ongoing education and training for healthcare workers in managing common medical emergencies like nosebleeds. A comparable study revealed that 85.9% of participants perceived a lack of public awareness about epistaxis first aid, while 89.6% agreed that first aid measures during epistaxis are crucial [15]. In the current research, participants' knowledge and attitudes towards nosebleed first aid management were not significantly different between genders, but age and academic years were significant factors. Younger participants and first-year students had lower knowledge scores. However, formal training in nosebleed first aid management significantly improved knowledge scores, regardless of demographics. Attitudes remained consistent across genders, ages, and academic years, suggesting targeted interventions were needed.

Other studies showed varying levels of knowledge by gender, with one reporting higher knowledge among men (94.7%) compared to women (37.6%) [16]. Participants aged 26–35 also demonstrated the highest knowledge levels in another study [16]. Al-Kubaisy *et al*. also investigated this connection but found no statistically significant differences in knowledge by demographic characteristics [17].

This study underscores the importance of enhancing knowledge and attitudes regarding first aid management of epistaxis among healthcare-related specialties students, with particular attention to the role of formal training in improving knowledge levels. By addressing gaps in understanding and fostering a proactive attitude towards nosebleed emergencies, healthcare professionals can better serve their communities and ensure prompt and effective care. Overall, our findings underline the significance of ensuring that medical students receive a thorough education on the distinctions between malpractice and surgical complications. This education should be an integral part of the medical curriculum and should be structured to ensure students can distinguish between these notions and comprehend their legal and ethical ramifications.

The present study has limitations that necessitate careful consideration when interpreting the results. The potential limitation of sample selection bias should be acknowledged, as the study exclusively targeted health-related specialty students and interns from a single university, which may restrict the applicability of the findings to a broader population. Additionally, using self-reported data obtained through surveys adds the possibility of recall bias and social desirability bias. Furthermore, using a cross-sectional design limits the ability to establish causal links and observe temporal changes. The dissemination of the questionnaire via social media platforms has the potential to generate selection bias. It is imperative for future research endeavors to acknowledge these limitations in order to effectively address the existing gaps and enhance comprehension of the awareness and attitudes of heath-related specialties students and medical interns towards epistaxis first aid.

## CONCLUSION

In conclusion, this study addresses knowledge gaps and enhances attitudes towards epistaxis first aid management among health-related specialty students at Al-Baha University in Saudi Arabia. Despite the prevalence of nosebleeds and their potential severity, there is a notable lack of documented understanding among students in this field. The findings underscore the need for targeted educational interventions to equip students with the necessary skills and knowledge to manage epistaxis incidents effectively. By bridging these gaps, healthcare professionals can better respond to nosebleed emergencies, ultimately improving patient outcomes and reducing the burden on healthcare systems. Implementing strategies that promote ongoing education and training in epistaxis management is imperative, ensuring that students are adequately prepared to handle such incidents in their future clinical practice.
